# Enantiomer-Selective Characterization of the Adsorption, Dissipation, and Phytotoxicity of the Plant Monoterpene Pulegone in Soils

**DOI:** 10.3390/plants11101296

**Published:** 2022-05-12

**Authors:** Jose Antonio Galán-Pérez, Beatriz Gámiz, Ivana Pavlovic, Rafael Celis

**Affiliations:** 1 Instituto de Recursos Naturales y Agrobiología de Sevilla (IRNAS), Consejo Superior de Investigaciones Científicas (CSIC), Avenida Reina Mercedes 10, 41012 Sevilla, Spain; jagalan@irnas.csic.es (J.A.G.-P.); rcelis@irnase.csic.es (R.C.); 2 Departamento de Química Inorgánica, Instituto Universitario de Investigación en Química Fina y Nanoquímica (IUIQFN), Universidad de Córdoba, Campus de Rabanales, 14071 Córdoba, Spain; ivana.pavlovic@uco.es

**Keywords:** allelochemicals, bioherbicides, monoterpenes, organoclays, phytotoxicity, soil

## Abstract

Plant monoterpenes have received attention for their ecological functions and as potential surrogates for synthetic herbicides, but very little is known about the processes that govern their behavior in the soil environment, and even less about the possible enantioselectivity in the functions and environmental behavior of chiral monoterpenes. We characterized the adsorption and dissipation of the two enantiomers of the chiral monoterpene pulegone in different soils, and their phytotoxicity to different plant species through Petri dish and soil bioassays. R- and S-pulegone displayed a low-to-moderate non-enantioselective adsorption on the soils that involved weak interaction mechanisms. Soil incubation experiments indicated that, once in the soil, R- and S-pulegone are expected to suffer rapid volatilization and scarcely enantioselective, biodegradation losses. In Petri dishes, the phytotoxicity of pulegone and its enantioselectivity to *Lactuca sativa*, *Hordeum vulgare*, and *Eruca sativa* was species-dependent. *Lactuca sativa* was the most sensitive species and showed higher susceptibility to S- than to R-pulegone. Biodegradation and volatilization losses greatly reduced the phytotoxic activity of S-pulegone applied to soil, but the addition of a highly-adsorptive organoclay stabilized the monoterpene and increased its phytotoxic effect. Stabilization by adsorption may represent an important mechanism by which the bioactivity of plant monoterpenes in soils can be increased.

## 1. Introduction

Allelopathy, the ecological phenomenon by which an organism affects neighboring organisms through the release of biochemicals (allelochemicals), represents an important environment-mediated plant–plant interaction mechanism [1,2]. Plant allelochemicals that reach the soil may directly affect seed germination, cell division, or seedling growth of target plant species, or impact abiotic or biotic soil processes with subsequent effects on the development of target plants [1,2,3]. In both cases, once in the soil, allelochemicals suffer adsorption, transport, and transformation processes which will ultimately dictate their bioactivity, and its changes with time [4,5,6,7].

Adsorption has been described as a key process determining the final fate of plant allelochemicals in the soil [8]. Depending on the mechanism, magnitude, and reversibility of the soil adsorption process, the activity of an allelochemical could decline if its fraction in the soil solution is not maintained at a bioactive level [9]. Transport and degradation losses can also reduce the concentration and bioactivity of allelochemicals in soils [10]. Very often, compounds that are highly phytotoxic under soilless laboratory conditions show much less activity in the presence of the soil, as a result of their rapid biodegradation [9,11,12]. In fact, the short half-lives that allelochemicals commonly display in soils, which prevents their accumulation in environmental compartments and subsequent effects on non-target organisms [13], represents one of the reasons why plant allelopathy has received the attention of the scientific community as an ecofriendly tool for weed management [9,14]. Allelochemical-based crop protection strategies can help overcome the negative impact of synthetic herbicides on the environment and human health, and deal with herbicide resistance problems [15,16]. Nevertheless, studies on the environmental factors and different interactions that regulate the behavior of allelochemicals in the soil have progressed slowly, and more work is needed to fill this knowledge gap [14,17].

Monoterpenes (C10 hydrocarbons derived from two C5 isoprene units) constitute a major group of plant allelochemicals [18]. They are volatile organic compounds dominating the composition of many plant essential oils [19,20], particularly in families such as *Pinaceae*, *Umbelliferae*, and *Lamiaceae* [21,22]. One of the most interesting facets of monoterpenes lies in their phytotoxicity, since they have been shown to be potent inhibitors of germination and growth of several plant species [23,24,25]. Monoterpenes that contain oxygen in their structure have greater water solubility and inhibitory activity than hydrocarbon monoterpenes [23,24,26,27], and, accordingly, have been particularly targeted for their ecological role and potential as surrogates for synthetic herbicides [26].

**Figure 1:**
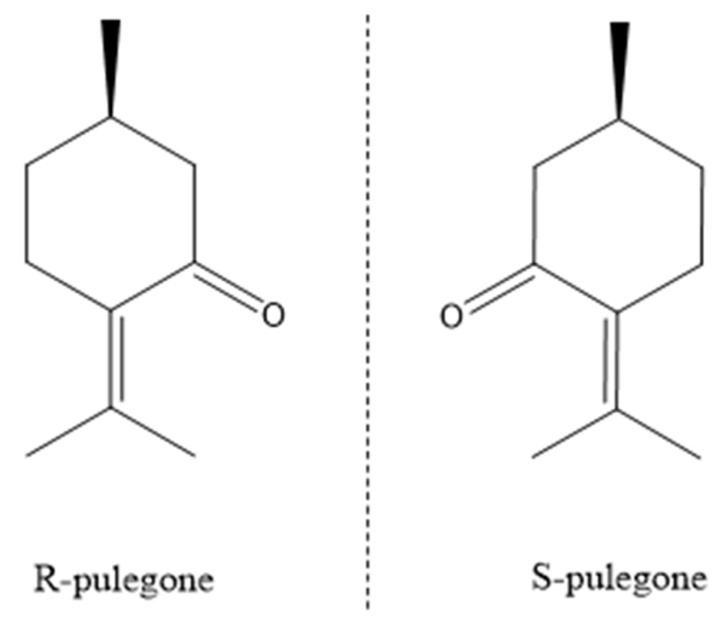
Structure of the two enantiomers of pulegone.

Pulegone (5-methyl-2-propan-2-ylidenecyclohexan-1-one) is an oxygenated monoterpene and one of the main components in essential oils from species of *Mentha* [28,29]. Different bioactivities have been described for pulegone, such as antifeedant; a biocidal against bacteria, fungi, and yeasts; and insect repellency activity [29,30,31,32]. Pulegone has also been shown to display a strong phytotoxic activity, suppressing germination and seedling growth of several plant species, such as *Lactuca sativa* and *Cucumis sativus* [23,24]. Very little is known, however, about the processes governing the behavior of pulegone in soils and about how soil processes can influence its phytotoxic activity. On the other hand, due to the presence of an asymmetric carbon in its structure, pulegone can exist as two enantiomers, R- and S-pulegone (Figure 1), with the R-enantiomer being more abundant in plants than the S-pulegone, but the latter predominating in the essential oils of some species, such as *Agathosma betulina* [33]. R-pulegone has been classified as “extremely active” and “strongly active” for germination and seedling growth of *Lactuca sativa*, respectively [24], and S-pulegone has been found to be less toxic to humans compared to R-pulegone [34]. Accordingly, the enantioselective behavior of this monoterpene should be considered, both to understand its ecological functions, and if intended to be used as a natural herbicide.

Given that monoterpenes are, in general, labile in the environment, their activities may be constrained by their susceptibility to volatilize [35] and by their short-half-lives in soils [6,31]. On the starting hypothesis that a deep understanding of the soil processes that dictate the behavior of monoterpenes in the soil environment will help a better assessment of their real ecological function and potential as ecofriendly herbicides, the objectives of this work were: (i) to characterize the adsorption and dissipation of the two enantiomeric forms of the monoterpene pulegone in soils with different physicochemical characteristics; (ii) to assess their phytotoxicity to different plant species through Petri dish and soil bioassays; and (iii) to demonstrate that reducing the dissipation losses of pulegone by enhancing the soil adsorption process can increase its phytotoxic activity and may represent a mechanism by which the bioactivity of monoterpenes in the soil environment can be potentiated.

## 2. Results and Discussion

### 2.1. Adsorption of Pulegone on the Soils and Model Soil Constituents

The adsorption coefficients for (R+S)-pulegone on the soils (K_d_), their organic carbon-normalized values (K_oc_), and the fraction of R-enantiomer (EF) measured in the aqueous phase after adsorption are compiled in Table 1. EF values in the range 0.497–0.503 revealed that the initial rac-pulegone solution (EF = 0.5) remained racemic after equilibration with the soils. This indicated that adsorption was a non-enantioselective process, with R-pulegone being adsorbed to the same extent as S-pulegone. Even though soils are known to contain chiral mineral and organic constituents with the potential to display selective affinities for enantiomeric forms of organic molecules [36,37], batch adsorption studies rarely reveal this phenomenon [38,39,40,41,42]. A possible explanation is that soil adsorption sites displaying no or little enantiomeric selectivity may predominate over enantiomer selective sites. An alternative explanation is that a high heterogeneity of soil adsorption sites could lead to global compensation of the enantioselective processes that might be occurring at the microscale, resulting in an apparent lack of enantioselectivity at the macroscopic scale at which adsorption is evaluated, using the batch equilibration procedure [43].

Since the adsorption of pulegone on the soils was non-enantioselective, the K_d_ values for the R- and S-pulegone enantiomers coincided with those given in Table 1 for (R+S)-pulegone. K_d_ values, ranging between 0.31 and 1.31 L/kg, indicated a low to moderate affinity of R- and S-pulegone for the soils and were comparable to those previously reported for the oxygenated monoterpene carvone in similar soil samples [6]. Linear correlations with soil properties (Supplementary Materials, Table S1) revealed a lack of statistically significant relationships between the K_d_ values and the silt, clay, carbonate, or pH of the soils, but significant correlations between the K_d_ values and the organic carbon (r = 0.735, *p* = 0.038) and sand (r = −0.766, *p* = 0.027) contents, even though these two soil properties were not significantly intercorrelated (r = −0.415, *p* = 0.306). As the negative relationship with the sand content of the soils implied a positive relationship with their silt + clay content, we concluded that organic matter (OM) together with the minerals present in the fine (silt/clay) fraction could both have been important in the adsorption of R- and S-pulegone by the tested soils. On the other hand, the extensive desorption of the enantiomers (% Des), measured in an extraction step conducted with methanol immediately after the adsorption experiment (Table 1), reflected that weak interaction mechanisms operated in the binding of R- and S-pulegone to the soils.

**Figure 2:**
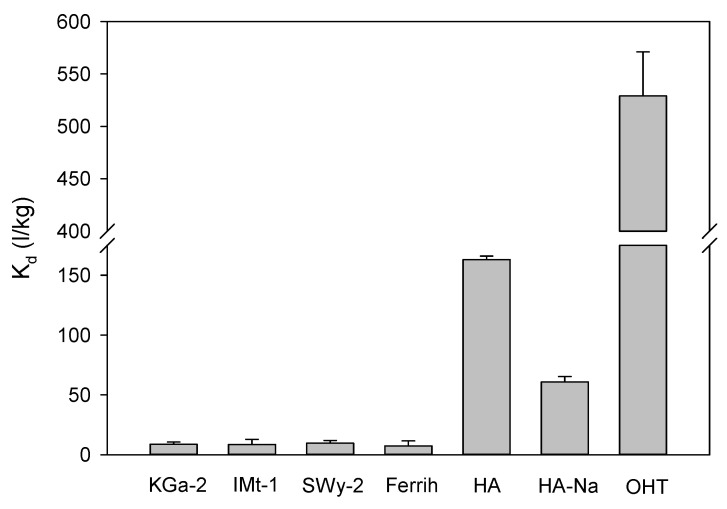
Distribution coefficients for rac-pulegone on model soil constituents and on OHT. KGa-2: Georgia kaolinite; IMt-1: Montana illite; SWy-2: Wyoming montmorillonite; Ferrih: ferrihydrite; HA: humic acid (acid form); HA-Na: humic acid (sodium salt); OHT: oleate-modified hydrotalcite.

Additional insight into the contribution of different soil constituents to the adsorption of R- and S-pulegone was obtained from the use of model soil constituents (Figure 2). As found for the soils, the adsorption of pulegone was non-enantioselective and, thereby, the K_d_ values reported in Figure 2 for (R+S)-pulegone accurately represented those corresponding to the individual R- and S-pulegone enantiomers. Interestingly, the K_d_ values obtained for the organic constituents (HA-acid form and HA-sodium salt, K_d_ = 61–163 L/kg) were about 10–20 times greater than those obtained for the mineral constituents (SWy-2, IMt-1, KGa-2, Ferrih; K_d_ = 7.4–8.8 L/kg). This supported the importance of soil OM as a major contributor to the adsorption of pulegone enantiomers, but also revealed that the role of soil minerals typically existing in the fine (silt/clay) fraction may become relevant in low OM content soils rich in silt and clay. Using an organic carbon to OM conversion factor of 1.7 to calculate the OM content, we obtained that the silt + clay content of the soils used in this study was between 20 (soil 4) and 90 (soil 2) times greater than their OM content (Table 1). This would explain a relevant contribution of the minerals present in the fine (silt/clay) fraction to the adsorption of pulegone enantiomers, as suggested by the outcomes of the correlation analysis.

### 2.2. Soil Dissipation

**Figure 3:**
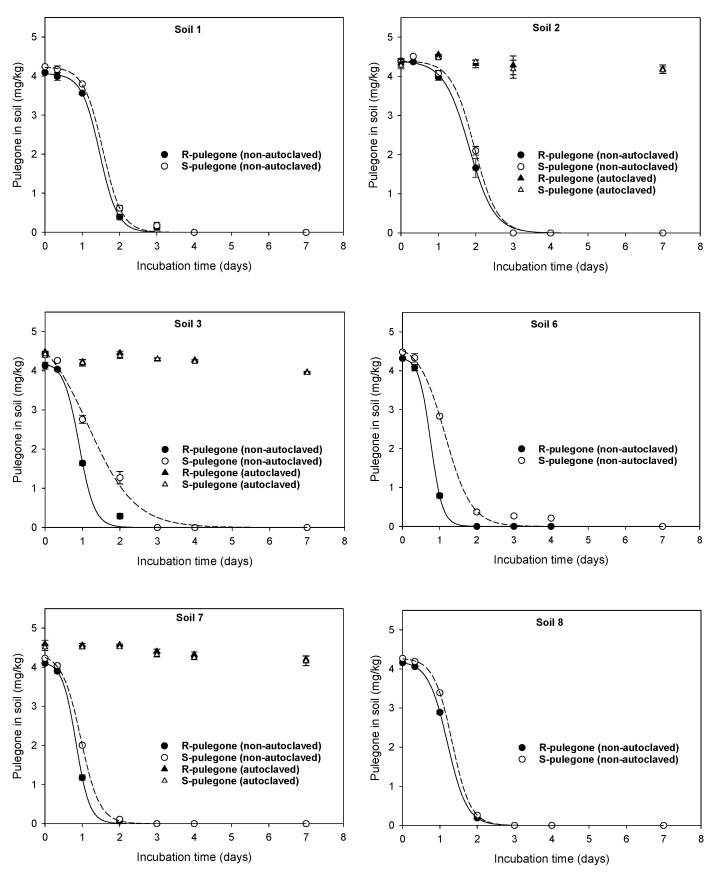
Dissipation patterns of R- and S-pulegone in non-autoclaved and autoclaved soils. Symbols indicate experimental data points whereas lines correspond to the fits of a sigmoidal three-parameter equation to the experimental data. Error bars denote standard errors of triplicate measurements.

Under conditions preventing volatilization losses (hermetically sealed tubes), R- and S-pulegone dissipated rapidly in non-autoclaved soils, while showing a high stability in autoclaved soils (Figure 3). This behavior was indicative that microbial processes, rather than non-microbial ones, governed the degradation of R- and S-pulegone in the soils. In all non-autoclaved soils, R- and S-pulegone dissipated, following sigmoidal curves, to negligible residual concentrations at the end of the experiment. Accordingly, a three parameter-sigmoidal equation (Equation (3)) accurately described the experimental dissipation data (R^2^ > 0.993) and was used to calculate the time required for the concentration of R- or S-pulegone to decrease to half of their initial concentration (DT_50_) (Figure 3, Supplementary Materials, Table S2). Sigmoidal dissipation patterns are typical of compounds at relatively high concentrations in soil that are utilized as energy and carbon sources by soil microorganisms [44].

The different properties of the soils had little impact on both the DT_50_ values of R- and S-pulegone (DT_50_ = 0.76–1.96 days) and the scarce enantioselectivity of the biodegradative processes. Nevertheless, the R-enantiomer appeared to be degraded faster than the S-enantiomer in some soils, particularly in the acid ones (Figure 3). In an earlier study, Gámiz et al. [6] compared the dissipation of the oxygenated monoterpene carvone in acid and alkaline soils, and assessed the effect of acidifying the alkaline soil on the rate and enantioselectivity of the dissipation process. Contrasting with the results obtained here for pulegone, soil acidity made the biodegradation of carvone slower and less enantioselective, which suggests that small differences in the structure of monoterpenes can significantly impact their biodegradation patterns in soil.

The effect of different factors on the dissipation of R- and S-pulegone in non-autoclaved soil 2 was also assessed (Supplementary Materials, Figure S1). The initial concentration, soil water content, temperature, and aeration all affected the dissipation rate. The effects were similar on both enantiomers, with a reduced dissipation rate at higher initial enantiomer concentration, at very high (40%), or limited (10%) soil humidity, and at low temperature (4 °C), probably reflecting the effect of these parameters on the activity of the soil microbial degraders. For example, under incubation conditions of 25 °C and 30% soil water content in sealed tubes, 3 days after treatment the residues of R- and S-pulegone applied at 5 mg/kg to the soil had become negligible. In contrast, they remained close to 70% of the amount initially added when the application dose was increased to 20 mg enantiomer/kg soil, to 25% when the soil water content was increased to 40%, and to 100% when the temperature was reduced to 4 °C (Supplementary Materials, Figure S1). Interestingly, a comparison of the dissipation patterns of S- and R-pulegone in open versus hermetically closed tubes revealed significantly higher losses of both enantiomers in the open tubes (Supplementary Materials, Figure S1). After 24 h, for example, losses increased from 10% in sealed tubes to 40% in open tubes. We attributed this effect to volatilization, which is known to represent a major dissipation route for monoterpenes in the environment [45,46].

### 2.3. Petri Dish Phytotoxicity Tests

The results obtained in the phytotoxicity tests of R- and S-pulegone to *Lactuca sativa*, *Eruca sativa*, and *Hordeum vulgare* conducted in Petri dishes are summarized in Table 2. The dose–response curves for the effect of each enantiomer on germination, root length, and shoot biomass of each plant species are detailed in Figure S2 of the Supplementary Materials. We observed that the phytotoxicity of pulegone and its enantioselectivity was species-dependent. For all germination and growth parameters studied, IC_50_ values increased in the order: *Lactuca sativa* ≤ *Hordeum vulgare* < *Eruca sativa* for both enantiomers (Table 2). *Eruca sativa* was the least sensitive species to R- and S-pulegone and displayed a characteristic hormetic response, by which R- and S-pulegone at concentrations around 100 mg/L stimulated the root growth (Supplementary Materials, Figure S2) [47,48]. This effect was not observed for *Lactuca sativa* or *Hordeum vulgare* (Supplementary Materials, Figure S2). On the other hand, the S-enantiomer of pulegone was noticeably more phytotoxic than the R-enantiomer to *Lactuca sativa* but not to the other two species. The lowest IC_50_ was observed for the effect of S-pulegone on the shoot biomass of *Lactuca sativa*, IC_50_ = 11 mg/L, a value 3 and 20 times lower than those obtained for *Hordeum vulgare* and *Eruca sativa*, respectively (Table 2). The activity of monoterpenes has previously been reported to be species-selective, even within the same family or genus [24], and enantiomer-selective phytotoxic effects have been observed for other chiral monoterpenes, such as carvone and citronellal [26].

### 2.4. Soil Bioassay: Effect of Oleate-Modified Hydrotalcite as a Soil Improver

**Figure 4:**
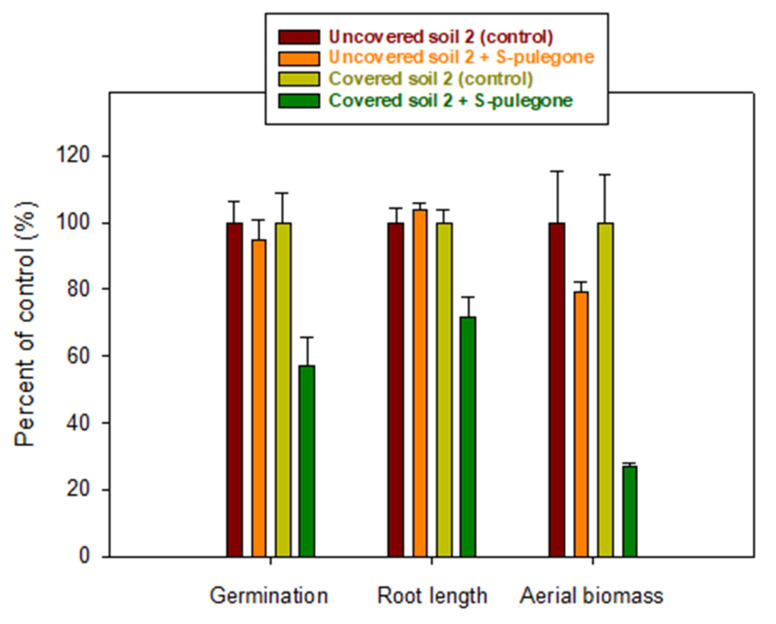
Effect of S-pulegone applied at 12 kg/ha on germination, root length, and aerial biomass of *Lactuca sativa* sown in pots filled with soil 2. The effect of covering the soil pots with plastic films to reduce volatilization losses of S-pulegone is shown in the graphs. Error bars correspond to the standard errors of triplicate measurements.

Based on the results of the bioassay conducted in Petri dishes, the S-enantiomer of pulegone and *Lactuca sativa* were selected as the best system to observe phytotoxic effects under soil conditions. The results of the phytotoxicity tests conducted with S-pulegone applied to soil 2 at a dose of 12 kg/ha are summarized in Figure 4. Considering the water content of the soil during the bioassay (30%), an application dose of 12 kg/ha corresponded to an initial aqueous concentration of 400 mg/L, i.e., the highest concentration tested in the Petri dish bioassay (Supplementary Materials, Figure S2). This concentration was 20 to 40 times greater than the IC_50_ values measured for S-pulegone on germination and seedling growth parameters of *Lactuca sativa* in the Petri dish bioassay (Table 2).

Figure 4 shows that the phytotoxicity of S-pulegone under soil conditions decreased drastically compared to that observed in the Petri dish study. Applied to soil pots, S-pulegone did not affect the germination or root growth of *Lactuca sativa*, and reduced only slightly the emerged seedling aerial biomass. According to the distribution coefficients reported in Table 1, adsorption was expected to reduce the bioavailable concentration of S-pulegone in the soil’s aqueous phase from 400 to 200 mg/L, a value still well above the IC_50_ of S-pulegone on *Lactuca sativa* germination and seedling growth (IC_50_ = 11–19 mg/L, Table 2). We concluded that rapid biodegradation and volatilization losses (Figure 3 and Figure S1) drastically reduced the phytotoxicity of S-pulegone applied to the soil. The effect of volatilization was evidenced by the finding that covering the soil pots with plastic films led to a significant increase in phytotoxicity (Figure 4). 

**Figure 5:**
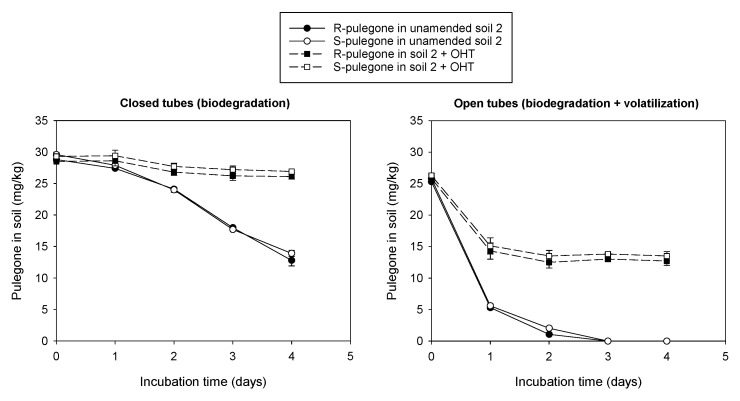
Dissipation curves of R- and S-pulegone in unamended and OHT-amended soil 2, measured in closed and open tubes. Error bars correspond to the standard errors of triplicate measurements.

**Figure 6:**
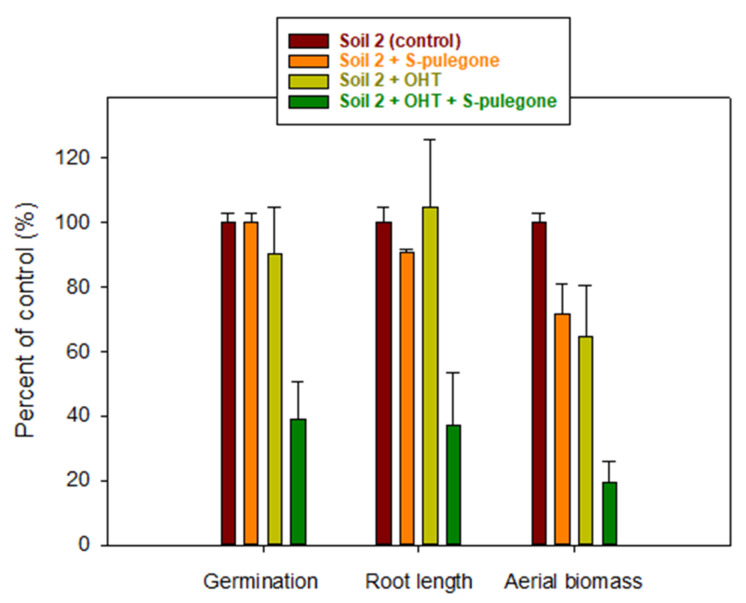
Effect of S-pulegone applied at 12 kg/ha on germination, root length, and aerial biomass of *Lactuca sativa* sown in non-covered pots filled with unamended and OHT-amended soil 2. Error bars correspond to the standard errors of triplicate measurements.

On the assumption that a reduction in the biodegradation and volatilization losses of S-pulegone could lead to a better expression of its phytotoxicity, we assayed the addition of a highly adsorptive organoclay material (OHT) as a strategy to stabilize the monoterpene in the soil through an enhancement in the adsorption process. Organically modified clays are known to be very good adsorbents of organic compounds [49] and have previously been successfully applied to increase the soil persistence of allelochemicals by protecting them from rapid leaching and degradation losses [50,51]. OHT was selected here for displaying a particularly high affinity for pulegone compared to similar organoclays (Supplementary Materials, Figure S3). The K_d_ value of pulegone on OHT (K_d_ = 525 L/kg, Figure 1) was three orders of magnitude greater than that on soil 2 (K_d_ = 0.32 L/kg, Table 1), whereby being applied at 1%, the material was expected to produce a 16-fold increase in the adsorption (K_d_) of pulegone by the soil. Figure 5 shows that the addition of OHT indeed reduced the biodegradation and volatilization losses of S-pulegone in soil 2, and Figure 6 shows how this resulted in an enhanced expression of its phytotoxicity in the soil.

## 3. Materials and Methods

### 3.1. Pulegone, Soils, Model Soil Constituents, and Adsorbent Material

R- and S-pulegone (Figure 1) were purchased as enantiopure compounds (purity > 98%) from Merck (Madrid, Spain). Pulegone enantiomers have a molecular mass of 152.2, a vapor pressure of 17 Pa at 25 °C [52], and a water solubility close to 400 mg/L [27]. The racemic (rac) aqueous solutions of pulegone used in the adsorption and dissipation experiments were obtained by diluting a stock rac-pulegone aqueous solution containing 200 mg/L of each enantiomer. The enantiopure pulegone aqueous solutions used in the bioassays were obtained from 400 mg/L individual stock solutions of R- or S-pulegone prepared in water.

Eight soil samples from southern Spain were selected for the study. All of them were collected from a 0–20 cm soil depth. Once in the lab, the soil samples were air-dried, sieved (2 mm), and kept at 4 °C until used. Their main properties are included in Table 1. The texture of the soil samples was determined by the hydrometer method [53], their carbonate content by the pressure calcimeter method [54], and their organic carbon content by dichromate oxidation [55]. The pH values were measured in 4 g:10 mL soil: water slurries.

The model soil constituents used were three phyllosilicates (SWy-2 montmorillonite, IMt-1 illite, and KGa-2 kaolinite), a two-line ferrihydrite (Ferrih), and two humic acids (HA-acid form and HA-sodium salt). The phyllosilicates were supplied by The Clay Minerals Society (CMS, Chantilly, VA, USA) and their properties are available at the CMS webpage [56]. The two-line ferrihydrite was prepared by precipitating a Fe(NO_3_)_3_ solution with NaOH, as described in Cruz-Guzmán et al. [57]. The humic acid samples were provided by Merck (Spain) and had organic carbon contents of 40.1% (HA-acid form) and 33.3% (HA-sodium salt).

The adsorbent material used as a soil modifier was an oleate-modified hydrotalcite (OHT), prepared by the reconstruction method commonly employed to obtain organo-hydrotalcites [49]. The preparation procedure is described in Gámiz et al. [40] and schematized in Figure S4 of the Supplementary Materials. Briefly, 50 g of commercial hydrotalcite (ref. 652288, Merck, Spain) was heated for 2 h at 500 °C, and then added to a solution containing 75 g of sodium oleate (ref. 26125, Merck, Spain) pre-dissolved in 1.5 l of water at a temperature of 60 °C. The suspension was stirred at 60 °C for 24 h and then filtered to recover the OHT solid sample. The analysis of OHT revealed a carbon content of 30.0% and a basal spacing value of 34.0 Å (Supplementary Materials, Figure S4).

### 3.2. Adsorption-Desorption Experiments

The adsorption of pulegone on the soil samples was measured in 14 mL-glass centrifuge tubes by equilibrating in an end-over-end shaker (24 h) 3 g of soil with 8 mL of a racemic solution of pulegone containing 1 mg/L of each enantiomer. In order to avoid biodegradation losses of pulegone, the soils were pre-autoclaved three times at 121 °C and 200 kPa for 20 min before the experiment [9]. After equilibration, the tubes were centrifuged (15 min at 2000× *g*) and 6 mL of the supernatant solutions were removed, stabilized with methanol (6 mL), filtered (GHP Acrodisc, 0.45 µm, Pall Corp. Madrid, Spain), and analyzed by HPLC to determine the individual concentrations of R-pulegone and S-pulegone remaining in solution. The amount of each enantiomer adsorbed by the soil was calculated from the difference between its initial and final concentration in solution. Control solutions (without soil) indicated that losses of the monoterpene as a result of processes other than adsorption to the soils were negligible. The adsorption of R- and S-pulegone on the model soil constituents and on OHT was measured following an identical methodology, but using 40 mg of adsorbent.

Desorption from the soils was evaluated by replacing the supernatant solution that was removed for the adsorption analysis with 8 mL of methanol. Subsequently, the tubes were re-equilibrated by shaking (24 h), centrifuging, filtering, and the concentrations of R- and S-pulegone in the extracts (C_ext_) were measured by HPLC. The amount of pulegone desorbed was expressed as a percentage of that previously adsorbed (% Des).

### 3.3. Soil Dissipation Experiments

The dissipation of pulegone in selected soils was studied through an incubation experiment conducted in 14 mL-glass centrifuge tubes, where 3 g of soil (non-autoclaved or autoclaved, according to the conditions detailed in Section 3.2) were treated with 0.9 mL of a solution of rac-pulegone containing 15 mg/L of each enantiomer. This yielded an initial (R+S) pulegone concentration in soil of 9 mg/kg and a soil humidity of 30%. Independent triplicate tubes were prepared, hermetically sealed, and incubated at 25 ± 1 °C in the dark to be removed from the incubator at selected times. Immediately after being removed, the tubes were frozen for subsequent extraction. For extraction, each tube was treated with methanol (8 mL), shaken for 24 h, centrifuged (15 min at 2000× *g*), and the supernatant was filtered and analyzed by HPLC to determine the concentration of R-pulegone and S-pulegone. This extraction procedure yielded recoveries >90% for the tested soils. For soil 2, we also evaluated the influence of the initial concentration of rac-pulegone (2, 9, or 40 mg/kg soil), the soil water content (40, 30, 10%), temperature/aeration (4 °C/non-aerated, 25 °C/non-aerated, 25 °C/aerated), and the addition of OHT at a rate of 1% on the pulegone enantiomers’ dissipation patterns. The methodology used for these treatments is detailed in the Supplementary Materials (Text S1).

### 3.4. Bioassays

We first compared the phytotoxicity of R- and S-pulegone to two dicot (*Lactuca sativa* and *Eruca sativa*), and one monocot (*Hordeum vulgare*) plant species, using a bioassay conducted in 9 cm-diameter Petri dishes. Each Petri dish contained one layer of filter paper as a support, 12 seeds of the dicots, or 9 seeds of the monocot, supplied by Vilmorin (France), and 6 mL of an enantiopure aqueous solution of R- or S-pulegone at concentrations between 0 (control) and 400 mg/L. Each treatment was conducted in triplicate Petri dishes, which were hermetically sealed and incubated in a germination chamber at 25 ± 2 °C, 70 ± 10% humidity, and 16:8-h light: dark photoperiod. After 5 days, we measured the number of germinated seeds, the radicle length for each seed, and the shoot biomass for each Petri dish, and expressed them as a percent of the values obtained for the control.

Based on the results obtained in the phytotoxicity tests conducted in Petri dishes, the S-enantiomer of pulegone and *Lactuca sativa* were selected as an optimum system to assess the phytotoxic activity of pulegone under soil conditions, as well as its changes by the addition of OHT as a soil modifier. The soil bioassay was conducted in plastic pots (60 cm^3^) containing 30 g of sea sand under a layer of 20 g of soil 2, either unamended or amended with 0.2 g of OHT. Triplicate soil pots were treated with 6 mL of a 400 mg/L solution of S-pulegone, to give an S-pulegone application dose of 12 kg/ha and a soil water content of 30%. Controls consisted of triplicates of unamended- or amended-soil pots treated identically, but without S-pulegone. Immediately after the application of the S-pulegone solution (or 6 mL of water for the controls), 12 seeds of *Lactuca sativa* were homogeneously distributed on the soil surface and the pots were incubated in the germination chamber for 7 days. During the experiment, the soil moisture content was re-adjusted daily to the value of 30%. At t = 7 days, the number of germinated seeds, and their root length and aerial biomass, were measured and expressed as a percentage of the values obtained for the control pots.

### 3.5. Analysis of Pulegone

The analysis of pulegone was carried out by HPLC using a Chiralpak IG column of 150 mm length and 4.6 mm internal diameter packed with amylose tris(3-chloro-5-methylphenylcarbamate) as a stationary phase (5 µm particle size), which allowed us to resolve the R and S enantiomers. The mobile phase was 50:50 (*v*:*v*) water: acetonitrile at a flow rate of 1 mL/min and quantification was carried out from the absorbance at 254 nm. Four standard solutions of rac-pulegone containing individual enantiomer concentrations between 0.1 and 6 mg/L in 50:50 (*v*:*v*) water: methanol as a solvent were used to obtain separate calibration curves for the R- and S-enantiomers of pulegone. The order of elution was elucidated by injecting enantiopure solutions of R-pulegone and S-pulegone under identical chromatographic conditions, which showed a retention time of 8.3 min for the R-enantiomer and 9.1 min for the S-enantiomer (Supplementary Materials, Figure S5).

### 3.6. Data Treatment

The adsorption distribution coefficients, K_d_, for pulegone (R+S) on the soils, model soil constituents, and OHT were determined as:

(1) $$K_{d}=\frac{C_{\mathrm{ads}}}{C_{\mathrm{aq}}}\;$$

where C_aq_ (mg/L) corresponded to the total (R+S) concentration of pulegone in solution after equilibration and C_ads_ (mg/kg) to the total amount (R+S) of pulegone adsorbed. To assess the possibility of preferential adsorption of one enantiomer over the other, the fraction of R-enantiomer (EF) in the equilibrated solutions was compared with that of the initial rac-pulegone solution (EF = 0.5) using the expression [58]:

(2) $$\mathrm{EF}=\frac{C_{\mathrm{aq}}\left(R\right)}{C_{\mathrm{aq}}\left(R\right){+C}_{\mathrm{aq}}\left(S\right)}\;$$

where C_aq_(R) and C_aq_(S) were the aqueous concentrations of R-pulegone and S-pulegone, respectively. Values of EF ≠ 0.5 would indicate the predominance of one enantiomer over the other in the aqueous phase after equilibration, and hence adsorption enantioselectivity.

Based on the dissipation patterns obtained for R- and S-pulegone in the soils, dissipation data were fit by the sigmoidal three-parameter equation available in SigmaPlot (v. 14.5, Berlin, Germany) for Windows:

(3) $$C=\frac{C_{0}}{{1+e}^{-\frac{t-{\mathrm{DT}}_{50}}{b}}}$$

where C (mg/kg) is the concentration of R- or S-pulegone in soil at time t (days), DT_50_ (days) is the time required for the concentration of R- or S-pulegone to decrease to half of their initial concentration (C_0_), and b defines the shape of the sigmoidal fit [59].

In the Petri dish bioassays, the three-parameter log-logistic equation of Sigma Plot 14.5 for Windows was used to perform a curve fit to the experimental data and to obtain the concentration of R- and S-pulegone needed to reduce the number of germinated seeds, radicle lengths, or shoot biomass to 50% of the control (IC_50_):

(4) $$y=\frac{a}{1+(\frac{x}{{\mathrm{IC}}_{50}}{)}^{b}}$$

where y and a represent the germinated seeds, radicle length, or shoot biomass (% of the control) at a specific concentration (x) of R- or S-pulegone (mg/L) and at x = 0, respectively [60].

Standard errors were employed to express variability among replicates. Bioassay data were subjected to one-way ANOVA and subsequent post hoc tests (Tukey’s HSD) for pairwise comparison between treatments. Significant differences were established at 95% confidence level.

## 4. Conclusions

At the macroscopic scale at which it was evaluated by the batch equilibration procedure, the adsorption of R- and S-pulegone by the soils and model soil constituents selected for the present study was a non-enantioselective process involving weak interaction mechanisms. Even though R- and S-pulegone displayed greater affinity for pure organic soil constituents than for mineral ones, the minerals present in the fine (silt/clay) fraction of the tested soils appeared to contribute significantly to the adsorption process, probably because of the low organic matter content of the soils. According to the soil K_d_ (0.31–1.31 L/kg) and K_oc_ (53–147 L/kg) values obtained, pulegone enantiomers can be classified as high to very high mobile compounds [61].

Once in the soil, R- and S-pulegone are expected to suffer rapid volatilization and microbial-mediated degradation losses rather than chemical degradation reactions. Soil biodegradation patterns strongly indicated the utilization of both enantiomers as energy and carbon sources by soil microorganisms. Although both R- and S-pulegone degraded quickly in all soils, the R-enantiomer appeared to be degraded even faster than the S-enantiomer in some soils, particularly in acid soils.

Under Petri-dish conditions, the phytotoxicity of pulegone and its enantioselectivity to different plants (*Lactuca sativa*, *Hordeum vulgare*, and *Eruca sativa*) was species-dependent. *Lactuca sativa* was the most sensitive species to pulegone enantiomers and displayed higher susceptibility to the S- than to the R-enantiomer. Soil bioassays showed that biodegradation and volatilization losses are likely to reduce considerably the phytotoxicity of S-pulegone under field conditions. However, enhancing the adsorption of S-pulegone through the addition of a highly adsorptive organoclay succeeded to reduce the biodegradation and volatilization losses and increase the activity of S-pulegone in soil. Stabilization by their adsorption on suitable matrixes may represent a promising tool to increase the bioactivity of monoterpenes in soil and could allow taking better advantage of their activities in their use as ecofriendly surrogates for synthetic pesticides.

## Figures and Tables

**Table 1:** Soil properties and coefficients for rac-pulegone adsorption.

Soil	Sand (%)	Silt (%)	Clay (%)	CaCO_3_ (%)	Organic C (%)	pH	K_d_ (L/kg)	K_oc_ ^1^ (L/kg)	EF ^2^	Des ^3^ (%)
1	83	6	11	6.4	0.35	8.5	0.31 ± 0.11	89	0.498	108 ± 25
2	74	4	22	0.6	0.17	8.0	0.32 ± 0.02	94	0.497	90 ± 6
3	66	23	11	0.7	0.99	5.2	1.02 ± 0.01	103	0.503	96 ± 7
4	63	19	18	17.9	1.18	8.4	0.63 ± 0.07	53	0.497	102 ± 5
5	57	24	19	3.6	0.85	8.3	0.73 ± 0.03	86	0.499	89 ± 7
6	55	36	9	n.d. ^4^	0.71	6.3	0.52 ± 0.02	73	0.501	106 ± 7
7	25	45	31	22.6	0.53	8.6	0.78 ± 0.03	147	0.498	90 ± 3
8	9	26	65	17.2	1.12	8.4	1.31 ± 0.03	117	0.498	67 ± 2

^1^ K_d_ value normalized to the organic carbon content of the soil; ^2^ Fraction of R-enantiomer in the equilibrated solution; ^3^ Percent of pulegone desorbed; ^4^ Not detected.

**Table 2:** Inhibitory concentrations (IC_50_) of R-pulegone and S-pulegone required to reduce germination, root length, and shoot biomass of *Lactuca sativa*, *Hordeum vulgare*, and *Eruca sativa* to 50% after 5 days of exposure in Petri dishes.

	IC_50 Germination_ (mg/L)		IC_50 Root Length_ (mg/L)		IC_50 Shoot Biomass_ (mg/L)	
	R-Pulegone	S-Pulegone	R-Pulegone	S-Pulegone	R-Pulegone	S-Pulegone
*Lactuca sativa*	60 ± 5	19 ± 1	37 ± 2	14 ± 1	25 ± 2	11 ± 1
*Hordeum vulgare*	55 ± 21	44 ± 9	56 ± 27	29 ± 7	35 ± 9	29 ± 6
*Eruca sativa*	312 ± 16	320 ± 21	-	-	257 ± 22	256 ± 17

## Data Availability

The data presented in this study are available in the article and the Supplementary Materials.

## Supplementary Materials

The following supporting information can be downloaded at: https://www.mdpi.com/article/10.3390/plants11101296/s1, Text S1: Description of the different treatments conducted to assess the effect of different factors on the dissipation of pulegone enantiomers in non-sterilized soil 2; Table S1: Pearson correlation coefficients between the pulegone K_d_ values on the soils and soil properties; Table S2: Parameters of pulegone dissipation data in non-autoclaved soils; Figure S1: Effect of the application rate, soil water content, and temperature/aeration on the dissipation of R- and S-pulegone in soil 2; Figure S2: Dose–response curves of R- and S-pulegone; Figure S3: Distribution coefficients for rac-pulegone on different organoclays; Figure S4: Schematic representation of the oleate-modified hydrotalcite sample (OHT); Figure S5: Chromatogram of a standard solution of rac-pulegone.
