# Supplementary material for: Enantiomer-Selective Characterization of the Adsorption, Dissipation, and Phytotoxicity of the Plant Monoterpene Pulegone in Soils
Source: Plants (Basel). 2022 May 12;11(10):1296. doi: 10.3390/plants11101296 (PMC9143748; doi:10.3390/plants11101296)
Supplement: Supplementary file 1 [file plants-11-01296-s001.zip › Supporting Information_Plants_12_05_2022.pdf]

Supplementary material

Enantiomer-selective characterization of the adsorption, dissipation, and phytotoxicity of the plant monoterpene pulegone in soils

Jose A. Galán-Pérez[1], Beatriz Gámiz[1,*], Ivana Pavlovic[2], Rafael Celis[1]

[1] *Instituto de Recursos Naturales y Agrobiología de Sevilla (IRNAS), CSIC, Avenida Reina Mercedes 10, 41012 Sevilla, Spain*

[2] *Departamento de Química Inorgánica, Instituto Universitario de Investigación en Química Fina y Nanoquímica (IUIQFN), Universidad de Córdoba, Campus de Rabanales, 14071 Córdoba, Spain*

***Corresponding Author:** B. Gámiz

Address: Instituto de Recursos Naturales y Agrobiología de Sevilla (IRNAS), CSIC

Avenida Reina Mercedes 10, 41012 Seville, Spain

E-mail: bgamiz@irnase.csic.es

Text S1. Description of the different treatments conducted to assess the effect of the application rate, soil water content, temperature/aeration, and the addition of OHT on the dissipation of pulegone enantiomers in non-sterilized soil 2.

i) Application rate: Triplicate samples of 3 g of soil were spiked with 0.9 mL of an aqueous solution of rac-pulegone at different concentrations (6, 30, or 150 mg L^{-1}) to give application rates of 2, 9 and 45 mg kg^{-1} soil and a soil water content of 30%. The samples were incubated in closed tubes at 25 ºC for 4 days.

ii) Soil water content: Triplicate samples of 3 g of soil were spiked with 0.3 mL of a 90 mg L^{-1} aqueous solution of rac-pulegone plus 0, 0.6 or 0.9 mL of water, to reach a rac-pulegone application rate of 9 mg kg^{-1} and soil water contents of 10, 30, or 40%, respectively. The samples were incubated in closed tubes at 25 ºC for 4 days.

iii) Temperature/aeration: Triplicate samples of 3 g of soil were spiked with 0.9 mL of a 30 mg L^{-1} aqueous solution of rac-pulegone to give an application rate of 9 mg kg^{-1} soil and a water content of 30%. The samples were incubated in closed tubes at 4ºC, closed tubes at 25ºC, or open tubes at 25ºC for 4 days. For the open tube-treatment the soil water content was re-adjusted daily to the initial value of 30%.

iv) Addition of OHT: Triplicate samples of 1 g of soil, either unamended or amended with 10 mg of OHT, were spiked with 0.3 mL of a 150 mg L^{-1} aqueous solution of rac-pulegone. The samples were incubated either in closed or open tubes at 25ºC for 4 days.

For all treatments, independent triplicate tubes were taken from the incubator at selected times (t= 0, 1, 2, 3, and 4 days), and frozen for subsequent extraction as described in the main text.

Table S1. Pearson correlation coefficients (r) between the pulegone K_d values on the soils (n = 8) and relevant soil properties. Statistically significant (P < 0.05) correlations are highlighted in bold.

Soil property	Pearson correlation coefficient (r)	*P* value
**Sand**	**-0.766**	**0.027**
Silt	0.468	0.242
Clay	0.690	0.058
$CaCO_3$	0.388	0.342
**OC**	**0.735**	**0.038**
pH	-0.168	0.690

Table S2. Paramaters resulting from fitting a sigmoidal 3-parameter equation to the R- and S-pulegone dissipation data in non-autoclaved soils.

Soil	R-pulegone				S-pulegone			
	C_0	b	DT_{50}	R^2	C_0	b	DT_{50}	R^2
1	4.06 ± 0.05	-0.240 ± 0.015	1.47 ± 0.03	1.000	4.23 ± 0.06	-0.257 ± 0.019	1.55 ± 0.04	0.999
2	4.39 ± 0.06	-0.337 ± 0.032	1.82 ± 0.03	0.999	4.39 ± 0.09	-0.306 ± 0.039	1.96 ± 0.06	0.998
3	4.22 ± 0.21	-0.205 ± 0.072	0.91 ± 0.05	0.996	5.18 ± 0.59	-0.621 ± 0.122	1.16 ± 0.21	0.993
6	4.36 ± 0.01	-0.160 ± 0.001	0.76 ± 0.01	1.000	4.70 ± 0.22	-0.353 ± 0.062	1.15 ± 0.06	0.996
7	4.15 ± 0.04	-0.183 ± 0.011	0.83 ± 0.01	1.000	4.34 ± 0.04	-0.251 ± 0.014	0.96 ± 0.01	1.000
8	4.20 ± 0.01	-0.260 ± 0.001	1.21 ± 0.01	1.000	4.28 ± 0.01	-0.246 ± 0.003	1.33 ± 0.01	1.000

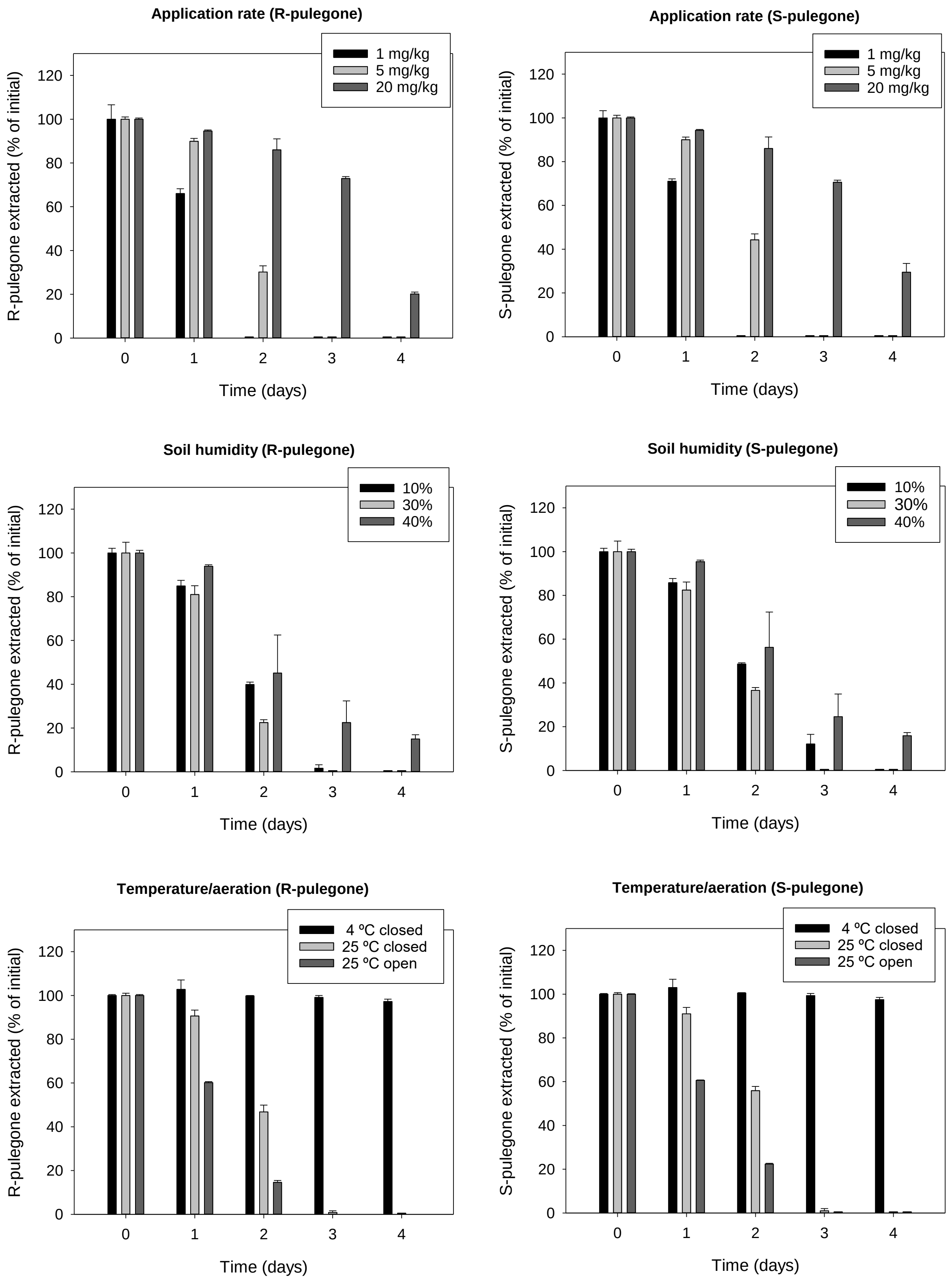

Figure S1. Effect of the application rate, soil water content, and temperature/aeration on the dissipation of R- and S-pulegone in soil 2. The different treatments are described in detail in Supplementary Text S1.

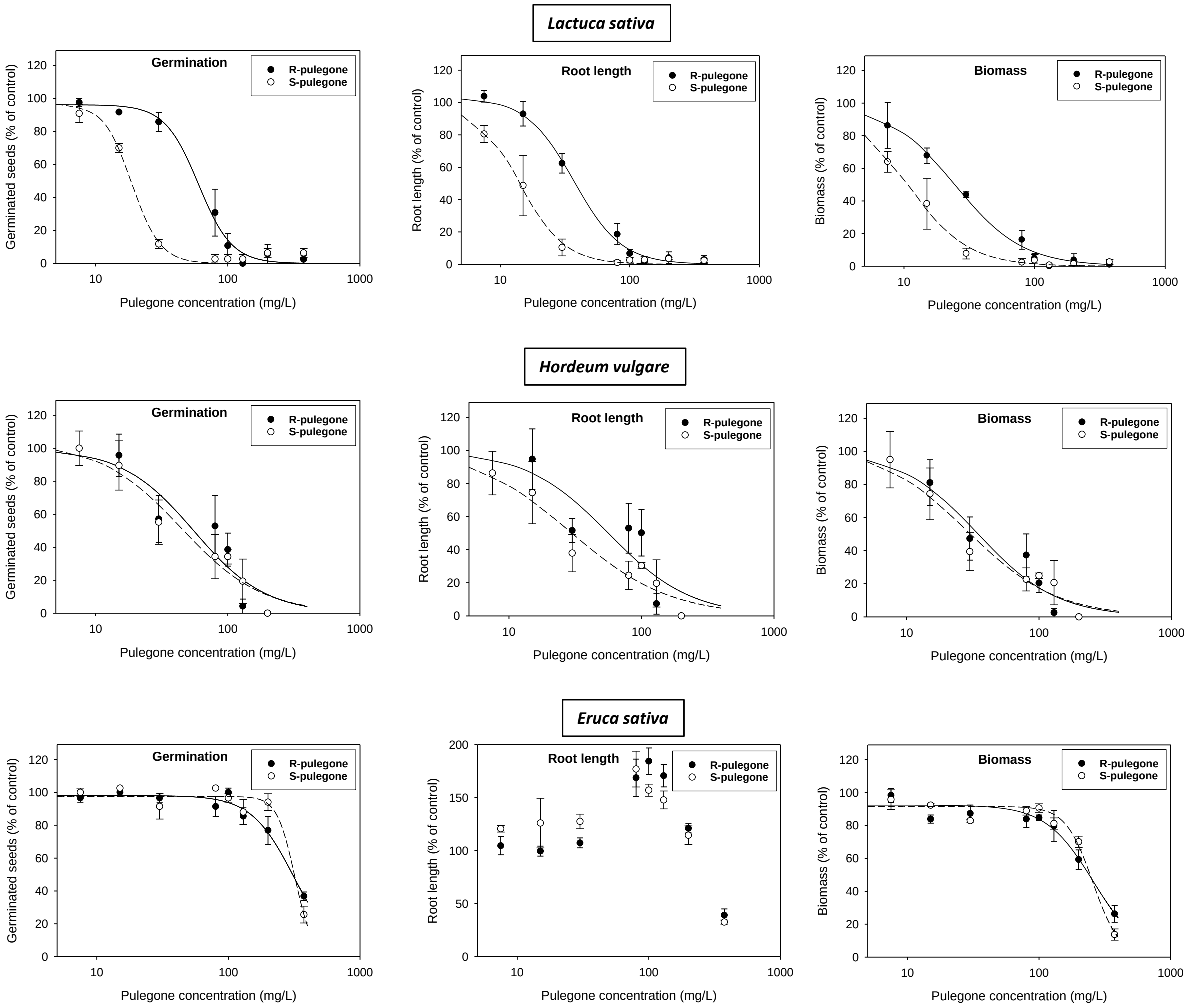

Figure S2. Dose-response curves of R- and S-pulegone on germination, root length, and shoot biomass of three plant species obtained in Petri dishes after 5 days. Symbols represent experimental percentages compared to the control, whereas lines are the log-logistic 3-parameter fits.

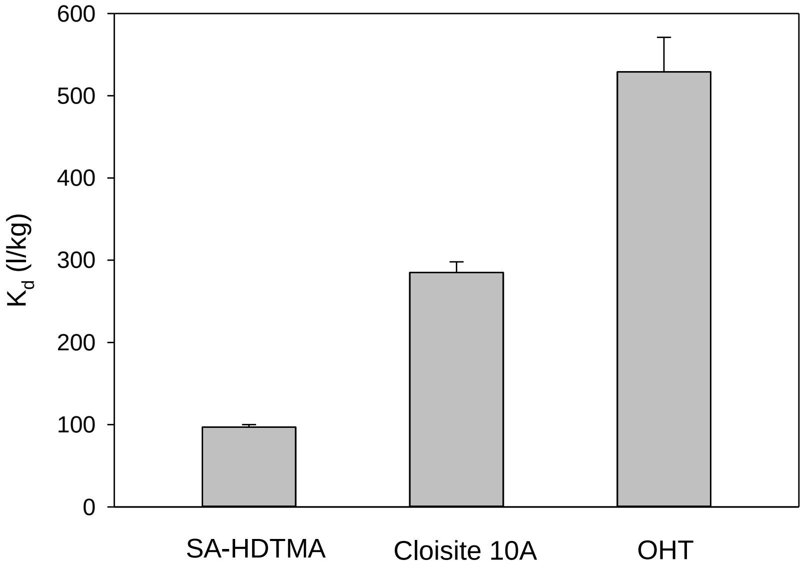

Figure S3. Distribution coefficients for rac-pulegone on different organoclays. SA-HDTMA: Arizona montmorillonite modified with hexadecyltrimethylammonium cations (lab-synthesized); Cloisite 10A: montmorillonite modified with dimethyl, benzyl, hydrogenated alkyl tallow quaternary ammonium cations (commercial); OHT: Oleate-modified hydrotalcite (lab synthesized). Measurements were conducted at an initial rac-pulegone concentration of 2 mg/l and an adsorbent to solution ratio of 40 mg:8 ml. Error bars denote standard errors of triplicates.

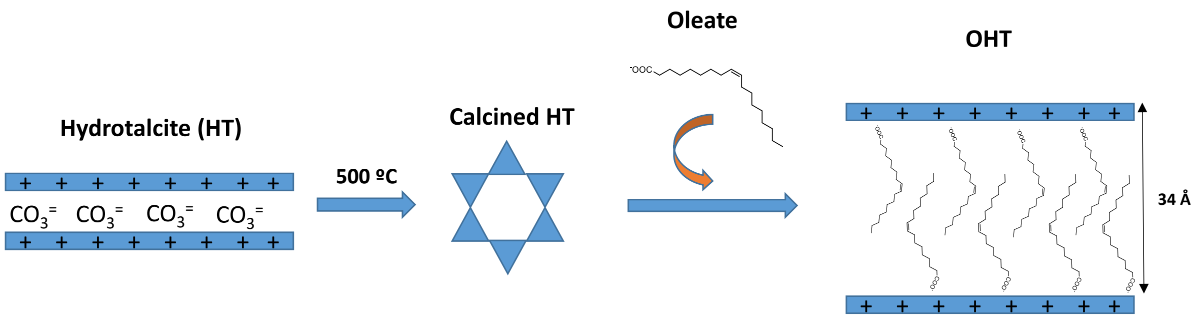

Figure S4. Schematic representation of the reactions leading to the formation of the oleate-modified hydrotalcite sample (OHT).

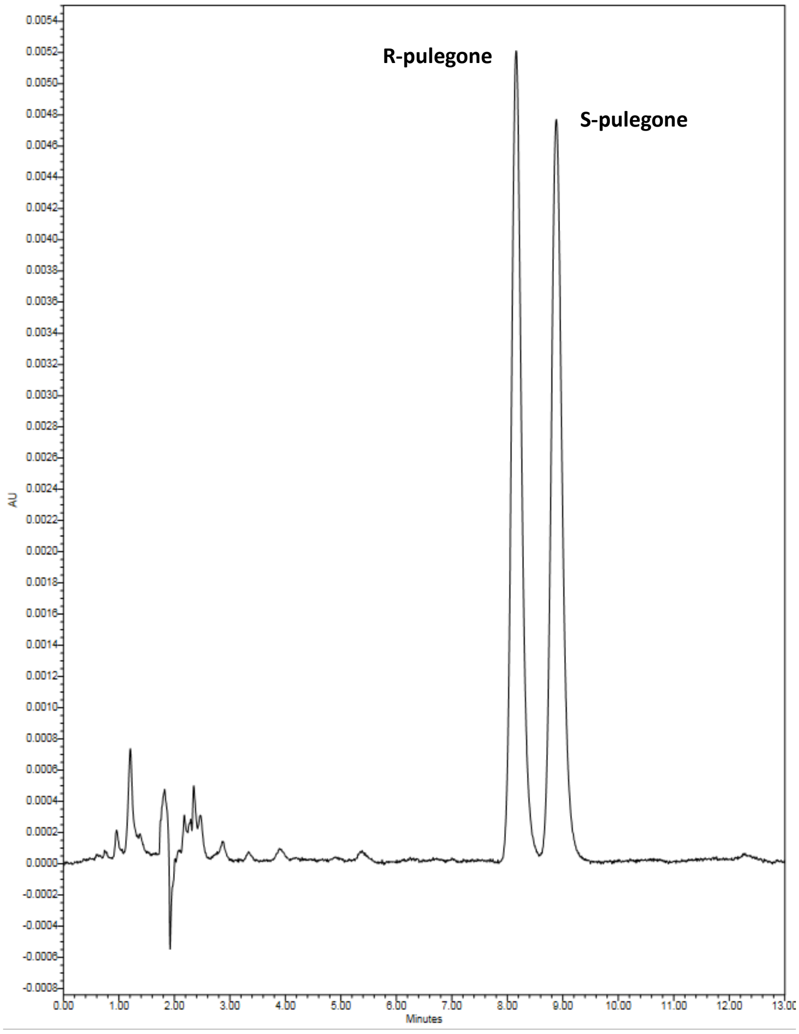

Figure S5. Chromatogram of a standard solution of rac-pulegone containing each enantiomer at a concentration of 1 mg/l in 50:50 methanol:water as a solvent.
